# Clinical factors affecting pathological fracture and healing of unicameral bone cysts

**DOI:** 10.1186/1471-2474-15-159

**Published:** 2014-05-17

**Authors:** Hiroshi Urakawa, Satoshi Tsukushi, Kozo Hosono, Hideshi Sugiura, Kenji Yamada, Yoshihisa Yamada, Eiji Kozawa, Eisuke Arai, Naohisa Futamura, Naoki Ishiguro, Yoshihiro Nishida

**Affiliations:** 1 Department of Orthopedic Surgery, Nagoya University Graduate School and School of Medicine, 65 Tsurumai, Showa-ku, Nagoya, Aichi 466-8550, Japan; 2 Department of Orthopedic Surgery, Aichi Cancer Center Aichi Hospital, 18 Kuriyado, Kake-machi, Okazaki, Aichi 444-0011, Japan; 3 Department of Orthopedic Surgery, Aichi Cancer Center Hospital, 1-1 Kanokoden, Chikusa-ku, Nagoya, Aichi 464-8681, Japan; 4 Department of Orthopedic Surgery, Nagoya Memorial Hospital, 4-305 Hirabari, Tenpaku-ku, Nagoya, Aichi 468-8520, Japan

**Keywords:** Unicameral bone cyst, Pathological fracture, Healing, Prognosis, Clinical factors

## Abstract

**Background:**

Unicameral bone cyst (UBC) is the most common benign lytic bone lesion seen in children. The aim of this study is to investigate clinical factors affecting pathological fracture and healing of UBC.

**Methods:**

We retrospectively reviewed 155 UBC patients who consulted Nagoya musculoskeletal oncology group hospitals in Japan. Sixty of the 155 patients had pathological fracture at presentation. Of 141 patients with follow-up periods exceeding 6 months, 77 were followed conservatively and 64 treated by surgery.

**Results:**

The fracture risk was significantly higher in the humerus than other bones. In multivariate analysis, ballooning of bone, cyst in long bone, male sex, thin cortical thickness and multilocular cyst were significant adverse prognostic factors for pathological fractures at presentation. The healing rates were 30% and 83% with observation and surgery, respectively. Multivariate analysis revealed that fracture at presentation and history of biopsy were good prognostic factors for healing of UBC in patients under observation.

**Conclusion:**

The present results suggest that mechanical disruption of UBC such as fracture and biopsy promotes healing, and thus watchful waiting is indicated in these patients, whereas patients with poor prognostic factors for fractures should be considered for surgery.

## Background

Unicameral bone cyst (UBC) is a benign bone lesion that affects children and young persons. This lesion has been reported together with aneurysmal bone cyst (ABC) [1]. USP6 and CDH11 oncogenes were reported to be identified in primary ABC [2], whereas the etiology of UBC remains unclear. Various pathogenetic mechanisms have been proposed including mechanical trauma, inflammation, and venous obstruction in the bone [3,4].

Because of the benign nature of UBC, the primary purpose of treatment is prevention of pathological fracture. Some reports have identified risk factors of pathological fractures. Previous reports indicated that high cyst index (area of the cyst/diaphysis diameter^2^) [5], high percent of bone occupied by the cyst in the transverse plane [6], thin cortical thickness [7], and location in the upper limb [8] significantly increased the incidence of pathological fractures. Although paradoxical, cyst healing was associated with the occurrence of pathological fractures. Previous reports showed that UBC healed spontaneously after fractures [6,9]. Other reports demonstrated that fracture [10], small cyst volume [10], cyst index [5], and long distance from the growth plate [11] significantly increased the rate of UBC healing after treatment.

The first aim of this study was to identify the risk factors associated with the occurrence of pathological fracture, since it is important to anticipate the risk of this complication when deciding on strategies of UBC treatment. The second purpose was to identify the factors influencing UBC healing, to aid in deciding treatment for patients with UBC.

## Methods

UBC patients who consulted 4 Nagoya musculoskeletal oncology group hospitals (Nagoya University Hospital, Aichi Cancer Center Hospital, Aichi Cancer Center Aichi Hospital, Nagoya Memorial Hospital) in Japan between January 1988 and February 2011 (n = 168) were collected. Thirteen patients were not suitable for the purposes of this study because of a lack of clinical records. One hundred fifty-five consecutive patients with UBC were retrospectively reviewed. As our treatment strategy of UBC, a biopsy was performed in patients in whom the possibility of other conditions was suspected (n = 30). Pathological fractures were treated as a rule with immobilization. Surgical treatments were carried out in symptomatic patients, cases predisposed to fracture, or for bone cysts located in the proximal femur in which dislocation of the fracture, coxa vera, and avascular necrosis of the femoral head were major concerns [12,13].

Baseline patient characteristics are summarized in Table 1. All 155 patients selected were evaluable for pathological fracture at presentation, and 141 patients with follow-up periods over 6 months were evaluable for UBC healing.

**Table 1:** Baseline characteristics of all patients (n = 155)

**Characteristics**	**Value (range) or No. of patients (%)**
Sex	
Male	101 (65%)
Female	54 (35%)
Age, years	
Median (range)	14 (2-58)
Size, cm	
Median (range)	4.0 (0.9-25.0)
Anatomic site	
Humerus	55 (35%)
Calcaneus	41 (26%)
Femur	35 (23%)
Pelvis	10 (6%)
Tibia	5 (3%)
Fibula	5 (3%)
Others	4 (3%)
Pathological fracture	
Yes	60 (39%)
No	95 (61%)

**Figure 1:**
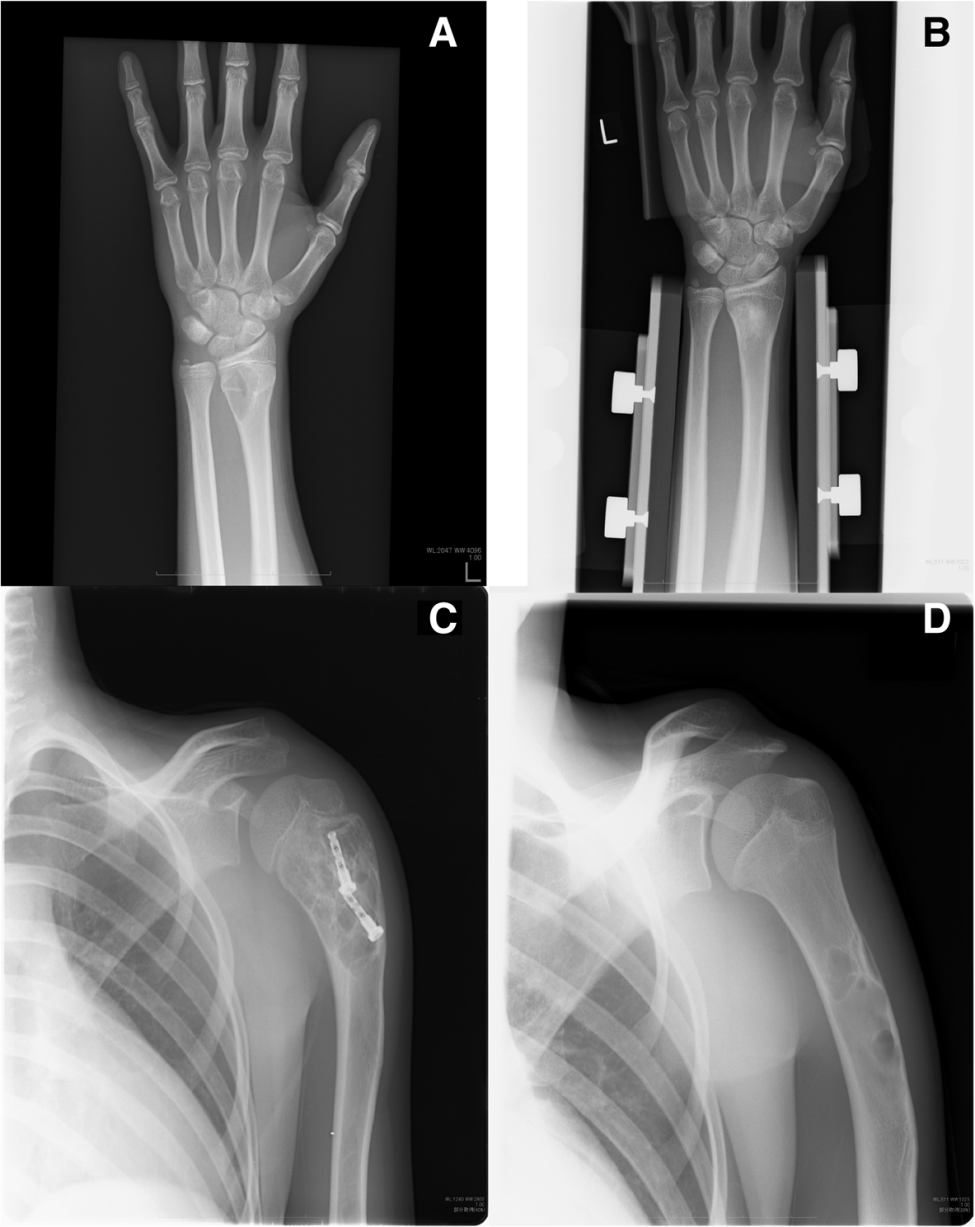
**Healing of UBC.** Fifteen-year-old male of UBC in his left distal radius: Anteroposterior radiograph at his first presentation **(A)** and 12 months later **(B)**. He had a pathological fracture at first visit and complete healing was observed after observation. Ten-year-old male of UBC in his left proximal humerus: Anteroposterior radiograph after surgery **(C)** and 4 years 7 months after surgery **(D)**. Partial healing was observed after surgery of cannulated screw.

Characteristics and treatments of 141 patients included in evaluation of healing are summarized in Table 2. The median follow up period was 28.7 (range 6.0-178.8) months. Seventy-seven cases were observed and 64 cases were treated by surgery. In patients of surgery, many of young patients with UBC were treated by cannulated screws, but various treatment methods were used for these patients. A biopsy was performed in 30 of 141 patients. Healing of the lesion was defined as healing of over 50% of the cyst area on X-ray at the last follow up (Figure 1).

**Table 2:** Characteristics and treatments of patients included in evaluation for healing (n = 141)

**Characteristics and treatments**	**Value (range) or No. of patients (%)**
Sex	
Male	90 (64%)
Female	51 (36%)
Age, years	
Median (range)	14 (2-58)
Size, cm	
Median (range)	4.0 (0.9-25.0)
Anatomic site	
Humerus	54 (38%)
Calcaneus	35 (25%)
Femur	31 (22%)
Pelvis	9 (6%)
Tibia	5 (4%)
Fibula	5 (4%)
Others	2 (1%)
Pathological fracture at first visit	
Yes	59 (42%)
No	82 (58%)
Biopsy	
Open	25 (18%)
Needle	5 (4%)
None	111 (79%)
Treatments	
Observation	77 (55%)
Surgery	64 (45%)
Operative procedure at first time	
Cannulated screws	35 (55%)
Curettage and graft	12 (19%)
Curettage	3 (5%)
Drilling	3 (5%)
Others	11 (17%)
No. of surgeries	
Single	50 (78%)
Multiple	14 (22%)
Follow-up, months	
Median (range)	28.7 (6.0-178.8)

Clinical data were collected from the patient’s clinical records. Chi-square test or Fisher’s exact test and multiple logistic regression were used to analyze the correlation of various clinical factors with fracture at presentation or healing of UBC. Clinical factors such as sex, age (≧20, <20), sites (long bone or not) (specific bone or not), diameter (≧5 cm, <5 cm), multilocular, ballooning of bone, and cortical thickness (≧2 mm, <2 mm) were analyzed for occurrence of fracture at presentation. In addition to these factors, fractures at presentation, history of biopsy, treatment (observation, surgery), and surgery (placement of cannulated screw or not) (single, multiple) were evaluated for healing of UBC. UBCs in humerus were classified into active phase (juxtaposed to the cartilaginous growth plate) or latent phase (migrating away and separating from the growth plate) [4], and the phase (active, latent) was evaluated for healing. *P*-values of <0.05 were considered to indicate significance.

This study was approved by Nagoya university ethics committee in November, 2012, and the registration number was 2012–0202. After obtaining a waiver of patient informed consent requirements from our institutional review board, we conducted a retrospective review from clinical records.

## Results

Sixty of 155 patients had pathological fracture at presentation. In univariate analysis, cyst in long bone (*p* < 0.001), diameter more than 5 cm (*p* < 0.001), multilocular cyst (*p* < 0.001), ballooning of bone (*p* < 0.001), cortical thickness less than 2 mm (*p* < 0.001), age less than 20 years (*p* = 0.001), and male sex (*p* = 0.006) were associated with a significantly increased incidence of pathological fractures at presentation (Table 3). Forty-three of 55 cases (78%) in humerus, 10 of 35 cases (29%) in femur, 2 of 10 cases (20%) in pelvis, 1 of 5 cases (20%) in tibia, 1 of 5 cases (20%) in fibula, 1 of 41 cases (2%) in calcaneus, and 2 of 4 cases in other bones had pathological fracture at presentation. In univariate analysis, UBC in humerus (43/55; 78%) had significantly more fractures than UBC at any other site (17/100; 17%) (*p* < 0.001), whereas UBC in calcaneus (1/41; 2%) had significantly less fracture than at any other site (59/114; 52%) (*p* < 0.001). In multivariate analysis, ballooning of bone (*p* = 0.011), cyst in long bone (*p* = 0.012), male sex (*p* = 0.013), cortical thickness less than 2 mm (*p* = 0.017), and multilocular cyst (*p* = 0.040) were significant risk factors for pathological fractures at presentation (Table 3). One patient with femoral neck UBC developed avascular necrosis after a displaced pathological fracture, and was treated by curved intertrochanteric varus osteotomy.

**Table 3:** Univariate and multivariate analysis of pathological fracture (n = 155)

**Clinical factors**	**No. of fractures (%)**	**Univariate analysis**	**Multivariate analysis**	
		***P*-value**^**a**^	***P*-value**^**b**^	**OR**^**c**^**(95%CI**^**d**^**)**
Sex				
Male	47/101 (47%)	*p* = 0.006	*p* = 0.013	3.13 (1.27-7.68)
Female	13/54 (24%)			
Age, years				
<20	53/114 (46%)	*p* = 0.001	n.s.	⁻
≧20	7/41 (17%)			
Site				
Long bone	55/100 (55%)	*p <* 0.001	*p* = 0.012	4.33 (1.39-13.54)
Others	5/55 (9%)			
Size, cm				
≧5	37/66 (56%)	*p <* 0.001	n.s.	⁻
<5	23/89 (26%)			
Multilocular				
Yes	39/66 (59%)	*p <* 0.001	*p* = 0.040	2.45 (1.04-5.76)
No	21/89 (24%)			
Ballooning				
Yes	49/79 (62%)	*p <* 0.001	*p* = 0.011	3.32 (1.31-8.37)
No	11/76 (14%)			
Cortex				
<2 mm	52/94 (55%)	*p <* 0.001	*p* = 0.017	3.36 (1.24-9.13)
≧2 mm	8/61 (13%)			

^a^chi-square test and Fisher’s exact test. ^b^multiple logistic regression. OR^c^, odds ratio. CI^d^, confidence interval.

Healing of UBC was less observed in the patients subjected to watchful waiting (23/77; 30%) than in operated patients (53/64; 83%) at the last follow up (*p* < 0.001). In observed patients, fracture at presentation (*p* = 0.004), multilocular cyst (*p* = 0.010), ballooning of bone (*p* = 0.015), and history of biopsy (*p* = 0.019) were significantly associated with good healing of UBC at the last follow up in univariate analysis (Table 4). Half of the patients (14/28 patients) with fracture at presentation and 57% of patients (8/14 patients) after biopsy showed healing with observation alone at the last follow-up. Five of these 28 patients were biopsied after fractures, with 4 of them showing healing at the last follow up. Multivariate analysis showed that fracture at presentation (*p* = 0.004) and history of biopsy (*p* = 0.013) were independent factors for good healing in observed patients (Table 4). Healing of UBC was observed in 26 of 35 patients (74%) after operation with cannulated screw, 12 of 12 patients (100%) with curettage and graft, 3 of 3 patients (100%) with curettage, 2 of 3 patients (67%) with drilling, and 10 of 11 patients (91%) with other methods at the last follow up. There was a statistical significant difference between cannulated screw and other treatment methods for healing of UBC (*p* = 0.046) (Table 5). UBC had healed in 40 of 50 patients (80%) after single surgery and in 13 of 14 patients (93%) after multiple surgeries (*p* = 0.244). In multivariate analysis for healing of UBC in humerus, latent phase UBC (*p* = 0.011) and treatment with surgery (*p* = 0.011) were significantly associated with good healing of UBC (Table 6). In patients with UBC in humerus, 3 of 10 patients with active phase and 12 of 21 patients with latent phase were healed at last follow up with observation (*p* = 0.152), and 6 of 11 patients with active phase and 12 of 12 patients with latent phase were healed after surgery (*p* = 0.014).

**Table 4:** Univariate and multivariate analysis for healing of UBC in observed patients (n = 77)

**Clinical factors**	**No. of healing (%)**	**Univariate analysis**	**Multivariate analysis**	
		***P*-value**^**a**^	***P*-value**^**b**^	**OR**^**c**^**(95%CI**^**d**^**)**
Sex				
Male	18/49 (37%)	*p* = 0.082	⁻	⁻
Female	5/28 (18%)			
Age, years				
<20	19/53 (36%)	*p* = 0.088	⁻	⁻
≧20	4/24 (17%)			
Site				
Long bone	17/50 (34%)	*p* = 0.281	⁻	⁻
Others	6/27 (22%)			
Size, cm				
≧5	12/31 (39%)	*p* = 0.164	⁻	⁻
<5	11/46 (24%)			
Multilocular				
Yes	13/27 (48%)	*p* = 0.010	n.s.	⁻
No	10/50 (20%)			
Ballooning				
Yes	15/34 (44%)	*p* = 0.015	n.s.	⁻
No	8/43 (19%)			
Cortex				
<2 mm	17/44 (39%)	*p* = 0.052	⁻	⁻
≧2 mm	6/33 (18%)			
Pathological fracture at first visit				
Yes	14/28 (50%)	*p* = 0.004	*p* = 0.004	5.31 (1.73-16.31)
No	9/49 (18%)			
Hisory of biopsy				
Yes	8/14 (57%)	*p* = 0.019	*p* = 0.013	5.47 (1.44-20.76)
No	15/63 (24%)			

^a^chi-square test and Fisher’s exact test. ^b^multiple logistic regression. OR^c^, odds ratio. CI^d^, confidence interval.

**Table 5:** Univariate analysis for healing of UBC after surgery (n = 64)

**Clinical factors**	**No. of healing (%)**	**Univariate analysis**
		***P*-value**^**a**^
Sex		
Male	33/41 (80%)	*p* = 0.386
Female	20/23 (87%)	
Age, years		
<20	42/53 (79%)	*p* = 0.103
≧20	11/11 (100%)	
Site		
Long bone	36/45 (80%)	*p* = 0.281
Others	17/19 (89%)	
Size, cm		
≧5	28/32 (88%)	*p* = 0.320
<5	25/32 (78%)	
Multilocular		
Yes	29/35 (83%)	*p* = 0.623
No	24/29 (83%)	
Ballooning		
Yes	32/40 (80%)	*p* = 0.341
No	21/24 (88%)	
Cortex		
<2 mm	36/45 (80%)	*p* = 0.299
≧2 mm	17/19 (89%)	
Pathological fracture at first visit		
Yes	26/31 (84%)	*p* = 0.828
No	27/33 (82%)	
Hisory of biopsy		
Yes	13/16 (81%)	*p* = 0.558
No	40/48 (83%)	
Surgery		
Placement of cannulated screw	26/35 (74%)	*p* = 0.046
Others	27/29 (93%)	

^a^chi-square test and Fisher’s exact test.

**Table 6:** Univariate analysis for healing of UBC in humerus (n = 54)

**Clinical factors**	**No. of healing (%)**	**Univariate analysis**	**Multivariate analysis**	
		***P*-value**^**a**^	***P*-value**^**b**^	**OR**^**c**^**(95%CI**^**d**^**)**
Sex				
Male	26/42 (62%)	*p* = 0.539	⁻	⁻
Female	7/12 (58%)			
Age, years				
<20	29/46 (63%)	*p* = 0.374	⁻	⁻
≧20	4/8 (50%)			
Phase				
Active	9/21 (43%)	*p* = 0.028	*p* = 0.011	0.16 (0.04-0.65)
Latent	24/33 (73%)			
Size, cm				
≧5	19/31 (61%)	*p* = 0.975	⁻	⁻
<5	14/23 (61%)			
Multilocular				
Yes	21/34 (62%)	*p* = 0.898	⁻	⁻
No	12/20 (60%)			
Ballooning				
Yes	28/41 (68%)	*p* = 0.055	⁻	⁻
No	5/13 (38%)			
Cortex				
< 2 mm	28/46 (61%)	*p* = 0.626	⁻	⁻
≧2 mm	5/8 (63%)			
Pathological fracture at first visit				
Yes	27/43 (63%)	*p* = 0.433	⁻	⁻
No	6/11 (55%)			
Hisory of biopsy				
Yes	8/12 (67%)	*p* = 0.461	⁻	⁻
No	25/42 (60%)			
Treatments				
				
Surgery	18/23 (78%)	*p* = 0.026	*p* = 0.011	6.90 (1.57-30.33)
Observation	15/31 (48%)			

^a^chi-square test and Fisher’s exact test. ^b^multiple logistic regression. OR^c^, odds ratio. CI^d^, confidence interval.

## Discussion

This is one of the largest studies to have focused on UBC in the trunk and extremities. There have been some clinical series of UBC including some with over 100 patients [1,14,15], but only a few large studies have been reported within the past quarter of a century [16].

Previous reports described some clinical factors that are associated with an increased incidence of pathological fracture with UBC. Radiographic features such as cyst index (area of the cyst/diaphysis diameter^2^) [5,7], percentage of bone occupied by the cyst in the transverse plane [6], and cortical thickness [7] were reported as prognostic factors of fractures with UBC. Another report showed that active phase UBC in an upper limb was at greater risk of fracture [8]. In our study, multivariate analysis revealed that ballooning of bone, cyst in long bone, male sex, thin cortical thickness and multilocular cyst had a significant impact on increasing the incidence of pathological fractures at presentation. There was a possibility that high activity in young males influenced the high occurrence of pathological fracture in our study. The high occurrence of fracture in ballooning bone, cysts in long bone and thin cortical thickness may reflect the vulnerability of ballooning bone and mechanical weakness of long bone, and multilocular UBC were usually observed in wildly spread cysts. In UBCs of humerus, latent phase significantly promoted good healing of UBC, with this finding consistent with previous reports [11]. These cysts at high risk for pathological fractures are clinically problematic and should be considered for surgery or careful observation.

Most of the pathological fractures were not displaced, were treated with immobilization, and healed without other complications, whereas one patient with femoral neck UBC in this study developed avascular necrosis after displaced pathological fracture. Some case reports have described avascular necrosis after pathological fracture in femoral neck UBC, and concluded that every effort should be made to prevent pathologic fractures because of the vascular supply to the upper femoral epiphysis in childhood and the reported incidence of avascular necrosis even with undisplaced fracture [12,13].

The clinical factors associated with good UBC healing have been reported as fracture [10], small cyst volume [10], rising cyst index [5], and long distance from the growth plate [4,11]. In our study, multivariate analysis showed that fracture at presentation and history of biopsy were good prognostic factors for healing of UBC in patients under observation, being partially consistent with the findings of previous reports. An especially interesting finding in our study was the effect of biopsy on healing of UBC. The diagnosis of UBC is usually not difficult, but some other lesions, especially in aged patients and patients after fracture, should be considered in the differential diagnosis such as primary or secondary ABC, fibrous dysplasia, non-ossifying fibroma, giant cell tumor, and osteosarcoma. A previous report showed that fractured UBCs frequently appear complicated on MR imaging, with heterogeneous fluid signals and regions of nodular and thick peripheral enhancement related to pathologic fracture and early healing [17]. Because some UBCs did not show typical imaging findings on X-ray or magnetic resonance imaging, we performed biopsy in 22% of patients. In these patients, 57% of UBC patients with watchful waiting had healed after biopsy, and a history of biopsy was noted to be an independent favorable factor for healing in observed patients.

In UBCs of humerus, latent phase significantly promoted good healing of UBC, with this finding consistent with previous reports [11]. All cysts with latent phase were healed after surgery, and cysts with latent phase at high risk for pathological fractures may be good indication for surgery.

Ahn et al. and Garceau et al. reported that 8% and 15% of UBCs healed after fractures, respectively [6,9], whereas in our study, 50% of UBCs after fracture healed without surgical treatment. Five of 28 patients with watchful waiting after fractures were biopsied, 4 of whom showed healing at the last follow up. The high biopsy rate in our study may have accounted for the high healing rate after fracture in the observed patients. The effect of pathological fracture and biopsy may be similar to that of making multiple holes in surgery [3].

In this study, UBC healing was defined as healing of the cyst area over 50% on X-ray at the last follow up. Many of UBC healings were observed in heterogeneous areas (Figure 1D), and the complete healing rate has been reported as being relatively low [18]. Since complete healing is not necessary for prevention of pathological fracture, we defined cyst healing over 50% as cyst healing.

Although various treatment methods have been proposed for UBC [15], less invasive methods such as cannulation, steroid injection, flexible intramedullary nailing, and endoscopic surgery have come to be preferred [19-22]. Elastic intramedullary nailing is the method of choice of treatment of unicameral bone cysts in children, it was reported to have the twofold benefits of continuous cyst decompression, and early immediate stability to the involved bone segment, which permits early mobilization and return to the normal activities of the preteen patients [22]. In our study, healing of UBC was observed in 74% of patients with cannulation and in 93% after other operations, while treatment with cannulated screw showed a lower healing rate than other surgical methods in univariate analysis. But this may not indicate the inferiority of this method for UBC. Tsuchiya et al. showed a higher success rate of 88% after treatment with cannulated screw [19]. The primary purpose of treatment in UBC is the prevention of pathological fracture, and the less marked invasiveness of cannulation is consistent with this purpose.

There were some limitations in our study. The first was the lack of data concerning the recurrence of UBC after treatment. We analyzed the final results of UBC healing at the last follow up after a median period of 28.7 (range 6.0-39.7) months, and did not take any recurrence of UBC into consideration. The recurrence rate of UBC was reported as 30% at the proximal end of the humerus and 17% at the proximal end of the femur [14]. Another report showed that the overall recurrence rate after surgery was 39% in a long follow up [23], although there was a further possibility of UBC relapse after the final last follow up. The second limitation was the evaluation of UBC healing in patients with multiple surgeries. Healing of UBC was observed in 53 of 64 cases treated surgically, although 13 of 53 patients healed only after multiple surgeries. We did not evaluate the effect of additional surgeries on UBC healing. Third, there was an apparent selection bias between treatment groups. The healing rates were 30% and 83% with observation and surgery, respectively, but a simple comparison was difficult to make because the two groups were not homogeneous. Finally, this study included various types of UBC, both pediatric and adult cases, long bone and others, and observed and operated cases. Especially this study included 41 of 155 cases of adult cases, but most of these were young adult and some of adult cases were pathologically diagnosed.

## Conclusions

In conclusion, ballooning of bone, cyst in long bone, male sex, thin cortical thickness and multilocular cyst were significant adverse prognostic factors for pathological fractures, whereas fracture at presentation and history of biopsy were favorable prognostic factors for healing of UBC in patients under observation. These clinical factors may be useful for deciding treatment in patients with UBC. A history of mechanical disruption of UBC such as fracture and biopsy seems to be good indication for watchful waiting, but patients with some poor prognostic factors of fractures or with UBC in the femur should be treated with surgery.

## Competing interests

All the authors declare that they have no financial and personal relationship with other people or organization that could potentially and inappropriately influence (bias) their work and conclusion.

## Authors’ contributions

HU, KH, NI, YN were involved in the conception and design of the study; ST, HS, KY, YY, EK, EA, and NF in acquisition of data; and HU and KH in analysis of data. HU drafted the article, and all authors edited and revised it for important intellectual content. HU, ST and YN take responsibility for the integrity of the work as a whole, from inception to finished article. All authors approved the finish version to be published.

## Pre-publication history

The pre-publication history for this paper can be accessed here:

http://www.biomedcentral.com/1471-2474/15/159/prepub
